# A Signature-Based Classification of Gastric Cancer That Stratifies Tumor Immunity and Predicts Responses to PD-1 Inhibitors 

**DOI:** 10.3389/fimmu.2021.693314

**Published:** 2021-06-11

**Authors:** Song Li, Jing Gao, Qian Xu, Xue Zhang, Miao Huang, Xin Dai, Kai Huang, Lian Liu

**Affiliations:** ^1^ Department of Medical Oncology, Qilu Hospital, Cheeloo College of Medicine, Shandong University, Jinan, China; ^2^ Department of Oncology, Affiliated Hospital of Shandong University of Traditional Chinese Medicine, Jinan, China

**Keywords:** gastric cancer, molecular classification, anti-tumor immunity, immune checkpoint inhibitors, tumor biomarkers

## Abstract

Gastric cancer is a leading cause of cancer-related deaths with considerable heterogeneity among patients. Appropriate classifications are essential for prognosis prediction and individualized treatment. Although immunotherapy showed potential efficacy in a portion of patients with gastric cancer, few studies have tried to classify gastric cancer specifically based on immune signatures. In this study, we established a 3-subtype cluster with low (C_LIM_), medium (C_MIM_), and high (C_HIM_) enrichment of immune signatures based on immunogenomic profiling. We validated the classification in multiple independent datasets. The C_HIM_ subtype exhibited a relatively better prognosis and showed features of “hot tumors”, including low tumor purity, high stromal components, overexpression of immune checkpoint molecules, and enriched tumor-infiltrated immune cells (activated T cells and macrophages). In addition, C_HIM_ tumors were also characterized by frequent *ARID1A* mutation, rare *TP53* mutation, hypermethylation status, and altered protein expression (HER2, β-catenin, Cyclin E1, PREX1, LCK, PD-L1, Transglutaminase, and cleaved Caspase 7). By Gene Set Variation Analysis, “TGFβ signaling pathway” and “GAP junction” were enriched in C_LIM_ tumors and inversely correlated with CD8^+^ and CD4^+^ T cell infiltration. Of note, the C_HIM_ patients showed a higher response rate to immunotherapy (44.4% vs. 11.1% and 16.7%) and a more prolonged progression-free survival (4.83 vs. 1.86 and 2.75 months) than C_MIM_ and C_LIM_ patients in a microsatellite-independent manner. In conclusion, the new immune signature-based subtypes have potential therapeutic and prognostic implications for gastric cancer management, especially immunotherapy.

## Introduction

Gastric cancer is a heterogeneous disease characterized by epidemiologic and histopathologic differences (1). Initially, it is histologically classified into either two subtypes (intestinal or diffuse) by the 1965 Lauren classification system or four subtypes (papillary, tubular, mucinous, or poorly cohesive) by the 2010 WHO system; yet, they both have limited implications on disease prognosis or treatment (2). In 2014, based on integrative analysis of multi-omics data, the TCGA group proposed four molecular subtypes: EBV-positive, microsatellite-instable (MSI), genomically stable, and chromosomal instability (3). In 2015, the Asian Cancer Research Group (ACRG) reported a similar classification based on mRNA expression profiles, including four subgroups associated with patients’ prognosis: microsatellite-stable (MSS)/TP53-, MSS/TP53+, MSI, and MSS/epithelial-to-mesenchymal transition (4). These classifications were proposed based on the characteristics of cancer cells, but alternative classification systems involving tumor microenvironments are seldom studied in gastric cancer.

Recently, immunotherapy, mainly represented by immune checkpoint inhibitors (ICIs), has shown promising efficacy in treating many types of cancers, including gastric cancer (5). In a phase III study ATTRACTION-2, nivolumab prolonged overall survivals of patients with ≥ two lines of treatment, leading to the approval of nivolumab in Asian countries (6). Similarly, previously treated advanced gastric cancer patients with PD-L1+ can benefit from pembrolizumab in the KEYNOTE-059 trial, achieving its approval in the United States (7). The CheckMate 649 trial demonstrated that the first-line treatment with nivolumab plus chemotherapy led to a statistically significant survival benefit compared to chemotherapy alone among gastric cancer patients with PD-L1 expression ≥5% (8). However, in phase III trials, KEYNOTE 062 (9) and ATTRACTION 04 (10), first-line treatment with ICIs plus chemotherapy failed to prolong the overall survival of advanced gastric cancer compared to chemotherapy alone. These data indicate an urgent need for biomarkers to predict responses and screen suitable patients who benefit from immunotherapy. Therefore, the classification of gastric cancer based explicitly on immune context may have clinical significance in guiding choices of immunotherapy.

In this study, we proposed and produced a feasible 3-subtype classification based on the immunogenomic profiling from three independent datasets and characterized molecular features at transcriptomic, proteomic, genetic, and epigenetic levels. This classification may have potential therapeutic and prognostic implications for gastric cancer management, especially immunotherapy.

## Materials and Methods

### Sample Datasets

**Figure 1:**
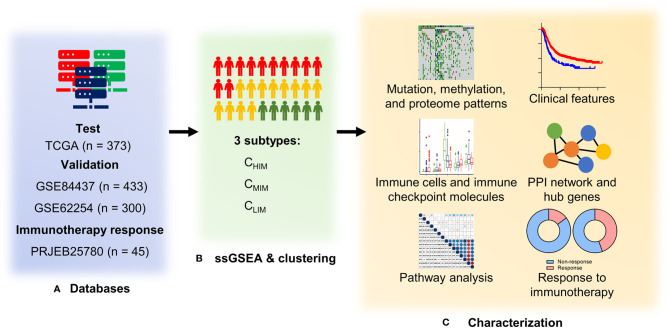
Study design and workflow of the present study. **(A)** Four databases of gastric cancer sequencing or gene array data were used as test and validation cohorts. **(B)** RNA expression data were quantified with 29 immune signatures by single-sample Gene Set Enrichment Analysis (ssGSEA) and hierarchically clustered into three subtypes. **(C)** Clinical features, genetic and epigenetic patterns, immune characteristics, and enriched pathways were compared among the three subtypes. In addition, an association between subtypes and responses to immunotherapy (pembrolizumab) was evaluated in the PRJEB25780 cohort.

Data from the training cohort, including level 3 data of RNA expression, somatic mutation (in the form of Mutation Annotation Format), and DNA methylation (based on the Illumina HumanMethylation 450 platform), of gastric cancer were downloaded from The Cancer Genome Atlas (TCGA) database (https://tcga-data.nci.nih.gov/tcga/). Gene methylation levels were estimated as β-values calculated as M/(M + U), where M represents signals from methylated beads, and U represents signals from un-methylated. Proteomic data of TCGA were downloaded from the Cancer Proteome Atlas (TCPA) databases (http://bioinformatics.mdanderson.org/main/TCPA). Data from the validation cohorts, including 733 gastric cancer patients in two datasets (GSE84437 and GSE62254) were downloaded from the Gene Expression Omnibus (GEO) database (https://www.ncbi.nlm.nih.gov/geo). Normalized RNA expression data of clinical cohorts with anti-PD-1 therapy (PRJEB25780) were downloaded from Tumor Immune Dysfunction and Exclusion (http://tide.dfci.harvard.edu/). Clinical data, including tumor shrink percent, MSI status, response, and progress-free survival, were extracted from the manuscript (11). The study design and workflow are outlined in **Figure 1**.

### Immune Signature-Based Subtype Classification

For training and validation cohorts, 29 immune signatures, including cell types, functions, and pathways, were used for subtype clustering. These signatures were derived from tumor tissue sequencing (12–18) and had been successfully used for tumor subtyping in several types of cancer (19–22). Immune signatures were quantified by the single sample Gene Set Enrichment Analysis (ssGSEA) algorithm by the R package “gsva” (http://www.bioconductor.org/packages/release/bioc/html/GSVA.html). Samples were hierarchically clustered into three subgroups with low (C_LIM_), medium (C_MIM_), and high (C_HIM_) immunity based on enrichment scores of ssGSEA. Gene compositions of each signature were listed in **Supplementary Table 1**.

### Survival Analyses

Overall survival of patients with specific tumor subtypes and expression profiles were compared using Kaplan-Meier curves and Logrank tests, with *p* < 0.05 as the significance threshold. Univariable and multivariable COX regressions were used to exclude confounding factors.

### Immune Score, Stromal Score, Tumor Purity, and Tumor-Infiltrating Analyses

Immune and stromal scores for quantification of immune/stromal cell infiltration were calculated by the Estimation of STromal and Immune cells in MAlignant Tumor tissues using the Expression data algorithm (ESTIMATE), utilizing specific gene expression signatures of immune and stromal cells (23). Relative frequencies of 22 different subsets of human immune cells in each tumor tissue were calculated by the CIBERSORT tool (24). Sample deconvolution was conducted using *P* < 0.05 and 1000 permutations as criteria. Kruskal-Wallis tests were used to compare the proportions of different immune cell subsets between tumor subtypes.

### Gene Set Variation Analysis

Gene Set Variation Analysis (GSVA) was used to estimate variations of pathway activity at single-sample levels in an unsupervised manner on RNA-seq data (25). It was performed using the R package “GSVA” downloaded at http://www.bioconductor.org.

### Differentially Expressed Gene Identification

Gene expression data from the TCGA database were used to identify differentially expressed genes (DEGs) between C_HIM_ and C_LIM_ samples by the R limma package. False discovery rate (FDR) values were calculated by the Benjamini-Hochberg method to correct *P*-values for multiple testing. DEGs were identified as genes with an FDR < 0.05 and a |log (fold change) | > 2.

### Construction of Protein-Protein Interaction Networks

STRING database (https://string-db.org/) was used to construct PPI networks with a 0.90 interaction cutoff. Cytoscape 3.7.2 (https://cytoscape.org/) was used to explore network topology. Genes with a degree of 10 or greater were considered hub genes.

### Statistics

Comparisons of categorical variables were investigated using Chi-square or *Fisher’s* exact tests. Comparisons of continuous variables among three groups were examined by ANOVA or Kruskal-Wallis tests. Comparisons of continuous variables between 2 groups were calculated by *t*-test. Benjamini-Hochberg methods were used to correct *P*-values for multiple testing. Survival curves were compared by Logrank regression. Univariable and multivariable COX regressions were used to measure the association between survival and other variables. Linear regressions were used for simple correlation. These statistical analyses were performed using Graphpad version 9.1 or R version 3.6.3. A *P*-value < 0.05 was considered statistically significant.

## Results

### Gastric Cancer Patients Are Classified Into Three Immune Signature-Based Subtypes

**Figure 2:**
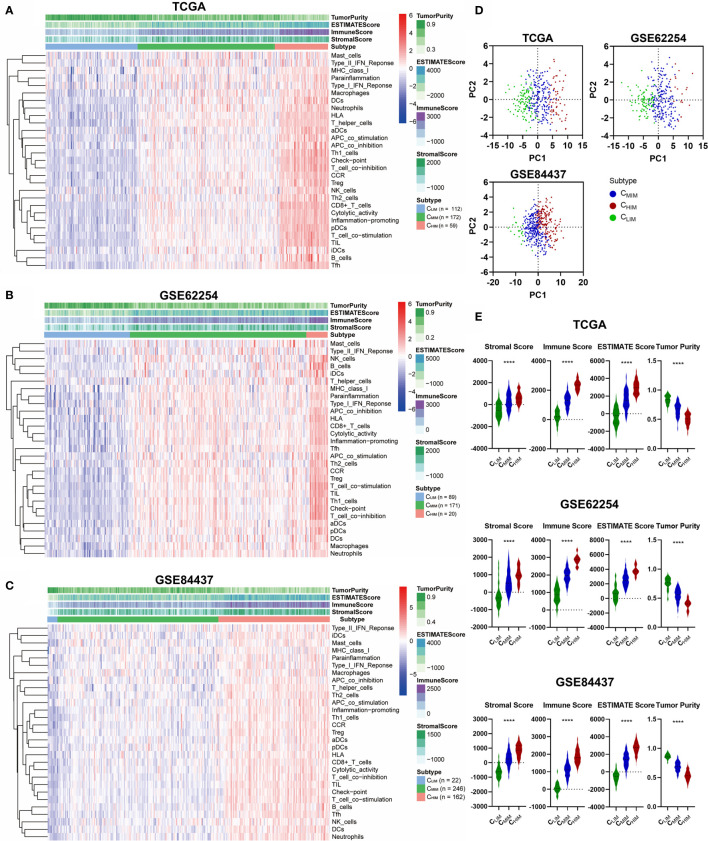
Hierarchical clustering of gastric cancer according to immune signatures. **(A–C)** Clustering of patients in TCGA **(A)**, GSE62254 **(B)**, and GSE84437 **(C)** datasets into 3 immune-based subtypes. **(D)** Principal component analysis of immune signature scores across the three databases. **(E)** Stromal scores, immune scores, ESTIMATE scores, and tumor purities of the three subgroups across three datasets. ****P < 0.0001.

We began by assessing the expression profiles of 29 immune-associated gene sets that correspond to specific signaling pathways, cell types, and functional activities, as previously reported (19–22). ssGSEA scores of these different gene sets in gastric cancer samples from three datasets, including TCGA, GSE62254, and GSE84437, were calculated, and hierarchical clustering was performed to group these samples into three subtypes. Similar clustering outcomes were achieved for all three datasets, with three separated sample clusters (**Figures 2A–C**). These clusters were defined as C_HIM_, C_MIM_, and C_LIM_, based on the relative expression levels of the 29 immune-associated gene sets. PCA analysis indicated that the subtypes were separated well from each other (**Figure 2D**).

By evaluating immune/stromal scores and tumor purity, we found that the C_HIM_ subtype contained higher numbers of immune cell infiltration, higher levels of stromal cells present, and lower tumor purity than non-C_HIM_ samples (**Figure 2E**). As expected, C_HIM_ tumors also had higher ESTIMATE scores (**Figure 2E**), a comprehensive evaluation of stromal cells and infiltrating immune cells (23).

### Immune Signature-Based Subtypes Exhibit Different Clinical Features and Survival Outcomes

We analyzed clinical features in these three subtypes across the three cohorts (**Supplementary Table 2**). In both TCGA and GSE62254 cohorts, immune subtypes had different EBV infection rates, which were the highest in C_HIM_ and the lowest in C_LIM_ (both *P* < 0.0001, **Supplementary Table 2**). In addition, distributions of patients with distant metastasis, deficient mismatch repair (dMMR), and *Helicobacter Pylori* infection were significantly different in GSE62254 (all *P* < 0.05, **Supplementary Table 2**).

To examine whether the three subtypes were correlated with patients’ survival, we first evaluated prognostic values of the 29 gene signatures by univariate COX regression analysis. The hazard ratio (HR) and 95% confidence intervals (CI) for the 29 gene sets are depicted in **Supplementary Figures 1A–C**. In the TCGA cohort, “NK cells”, “Inflammation promoting”, and “MHC class I”, were significantly associated with prolonged survival, whereas “T helper cells” and “Type II IFN response” were associated with poor outcome (**Supplementary Figure 1A**). However, these data were not consistent with those in the GSE62254 and GSE84437 cohorts, which indicated that one single signature alone was not powerful enough for survival prediction (**Supplementary Figures 1B, C**).

**Figure 3:**
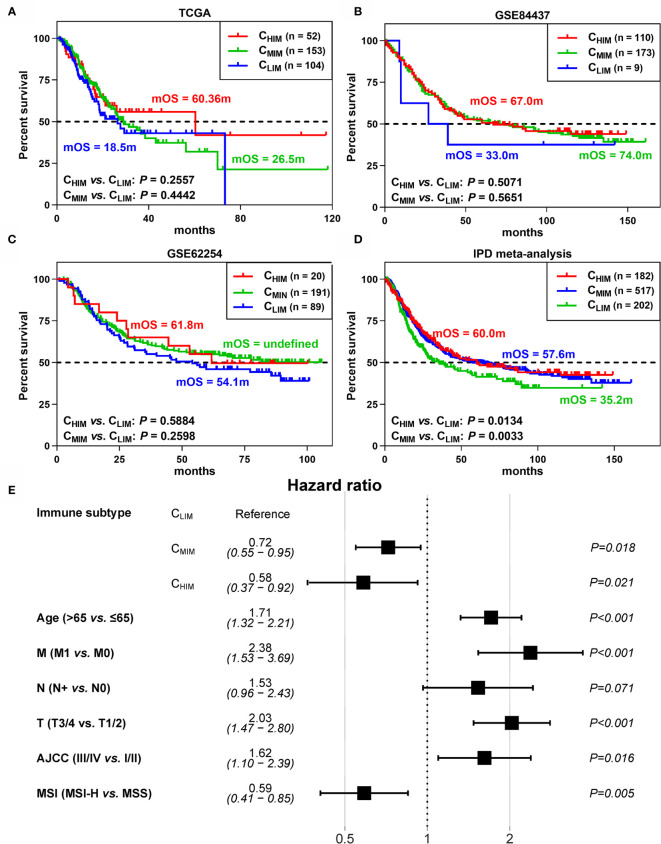
Differences in survival between immune subtypes. **(A–C)** Kaplan-Meier curves of survival data in patients of 3 subgroups from TCGA **(A)**, GSE62254 **(B)**, and GSE84437 **(C)** datasets. **(D)** Kaplan-Meier curves of survival data in the combined cohort by individual patient data (IPD) meta-analysis. **(E)** Hazard ratios of clinical factors by multivariable COX regression.

We next explored prognostic differences between the subtypes. In the three cohorts, the C_HIM_ and C_MIM_ patients showed prolonged median overall survivals (mOS) compared to the C_LIM_ subtype, with 60.4m and 26.5m *vs.* 18.5m in TCGA, 67.0m and 74.0m *vs.* 33.0m in GSE84437, and 61.8m and undefined *vs.* 54.1m (**Figures 3A–C**). However, HRs between these subgroups were not statistically significant (**Figures 3A–C**). To develop a conclusion with greater statistical power, we performed a meta-analysis based on individual patient data. Then the C_HIM_ and C_MIM_ groups showed prolonged mOS (60.0m and 57.6m *vs.* 35.2m) and low risk of death (HR 0.71 and 0.70, 95% CI 0.52-0.95 and 0.55-0.91) over the C_LIM_ subtype (**Figure 3D**). To exclude the confounding factors, we further performed univariable COX regression with immune subtypes (HR 0.69, 95% CI 0.52-0.92 for C_HIM_ and HR 0.70, 95% CI 0.56-0.89 for C_MIM_), age (HR 1.34, 95%CI 1.11-1.63), gender (HR 0.85, 95%CI 0.70-1.03), distant metastasis (HR 2.86, 95%CI 1.89-4.33), lymph node metastasis (HR 2.14, 95%CI 1.57-2.91), tumor size (HR 1.74, 95%CI 1.38-2.19), AJCC stage (HR 1.61, 95%CI 1.44-1.8), *Helicobacter Pylori* infection (HR 0.71, 95%CI 0.46-1.1), EBV infection (HR 0.84, 95%CI 0.50-1.42), MSI (HR 0.54, 95%CI 0.38-0.77), and race (HR 0.81, 95%CI 0.58-1.14). Then the factors with statistical significance were collected for multivariable COX regression (**Figure 3E**). C_HIM_ (HR 0.58, 95%CI 0.37-0.92) and C_MIM_ (HR 0.72, 95%CI 0.55-0.95) still showed better outcomes than C_LIM_ patients by multivariable COX regression.

### Immune-Based Subtypes Show Different Patterns Of Mutation, DNA Methylation, and Protein Expression

**Figure 4:**
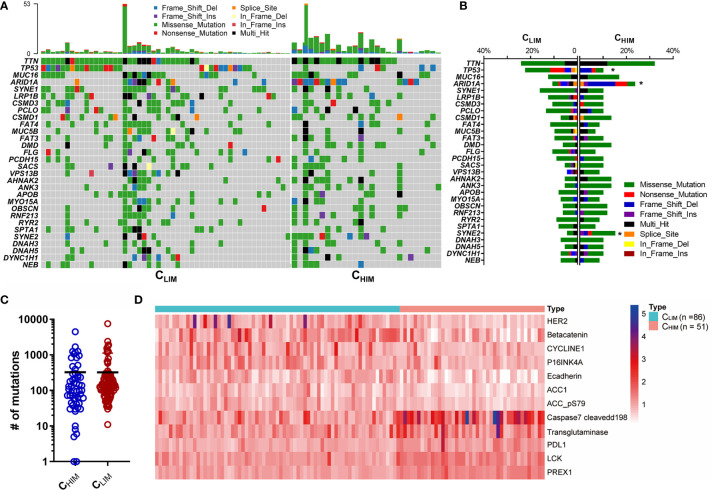
Genomic and proteomic features of different immune subtypes. **(A)** Oncoprint of gene mutations in C_HIM_ *vs.* C_LIM_ subtypes in the TCGA cohort. The top 30 mutated genes are shown. **(B)** Frequencies of mutations of the top 30 mutated genes in C_HIM_ *vs.* C_LIM_ patients. **(C)** Tumor mutation burden of the C_HIM_ and C_LIM_ patients in the TCGA cohort. **(D)** Differentially expressed proteins between C_HIM_ and C_LIM_ subgroups. *P < 0.05.

To investigate the comprehensive molecular characteristics among the 3 immune subtypes, we analyzed genomic mutation, DNA methylation, and proteomics data from the TCGA database. We found that most differences were more significant between C_HIM_ and C_LIM_, and C_MIM_ was at intermediate levels (**Supplementary Figures 2A–C**). The 30 most frequently mutated genes were presented in **Figure 4A**, and their mutation rates were compared in **Figure 4B**. Of note, *ARID1A* occurred in 23.7% of C_HIM_ patients but 10.7% of C_LIM_ patients, with a significant difference (**Figure 4B**). Meanwhile, mutations of *TP53* occurred more frequently in C_LIM_ than those in C_HIM_ patients (**Figure 4B**). Generally, the C_HIM_ group showed a comparable mutation burden to the C_LIM_ group (324.4 *vs.* 322.5, *P* = 0.9884, **Figure 4C**).

A recent study showed that high DNA methylation was associated with immune activation status, increased tumor mutation and neoantigens, and favorable prognoses in gastric cancer (26). In accordance with their finding, most differentially methylated genes were high in the C_HIM_ subtype (**Supplementary Figure 2C**), indicating a high-methylation status in this subgroup.

We also compared expression levels of protein derived from the RPPA data (**Figure 4D**). Seven proteins, including HER2, β-catenin, and Cyclin E1, were downregulated, while five genes, including PREX1, LCK, PD-L1, transglutaminase, and cleaved caspase-7, were overexpressed in the C_HIM_ subtype compared to in the C_LIM_ subtype.

### C_HIM_ Are Enriched With Anti-Tumor Immune Cells and Immune Checkpoints

**Figure 5:**
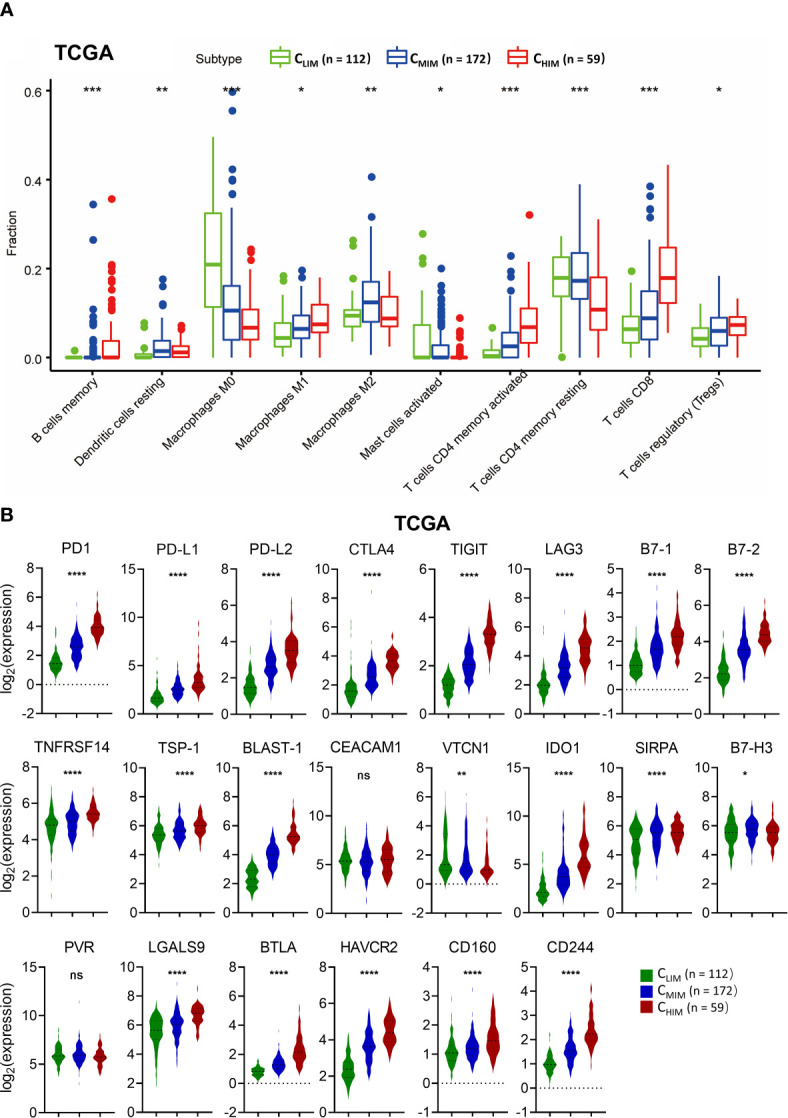
Comparison of immune cell infiltration and immune checkpoint molecule levels in different immune subtypes. **(A)** Fractions of tumor-infiltrated immune cells in three immune subtypes in the TCGA cohort. Only cells with significant differences among the subtypes are shown. **(B)** Levels of co-inhibitory molecules in three immune subtypes in the TCGA cohort. ^ns^ not significant, *P < 0.05, **P < 0.01, ***P < 0.001, ****P < 0.0001.

Tumor-infiltrating immune cells are often found within tumors and correlated with prognosis. We utilized the CIBERSORT algorithm to examine differences in immune cell infiltration patterns among the three clusters (**Figure 5A** and **Supplementary Figures 3A, B**). In the TCGA cohort, C_HIM_ samples had higher levels of anti-tumor cells, including CD8^+^ T cells, M1 macrophages, and CD4^+^ activated memory T cells, than the other two subtypes, whereas they had lower frequencies of M0 macrophage and CD4^+^ resting memory T cells (**Figure 5A**). These differences were also confirmed in the GSE84437 cohort (**Supplementary Figure 3A**). Besides, the enriched CD8^+^ T cells and depleted M0 macrophages in C_HIM_ were also observed in the GSE62254 cohort (**Supplementary Figure 3B**).

We also evaluated expression of immune checkpoints, some of which had been used as targets of immunotherapy. Most of the immune checkpoint molecules, including PD1, PD-L1, PD-L2, CTLA-4, LAG3, TIGIT, and TIM-3, were higher in C_HIM_ samples than in non-C_HIM_ samples (**Figure 5B**). These differences were consistent across TCGA (**Figure 5B**), GSE62254 (**Supplementary Figure 4A**), and GSE84437 (**Supplementary Figure 4B**). These findings indicate that the C_HIM_ tumors are clinically potential “hot tumors” that respond well to ICIs.

### Pathways Related to Immune Subtypes and Correlated With Infiltrated Immune Cells

**Figure 6:**
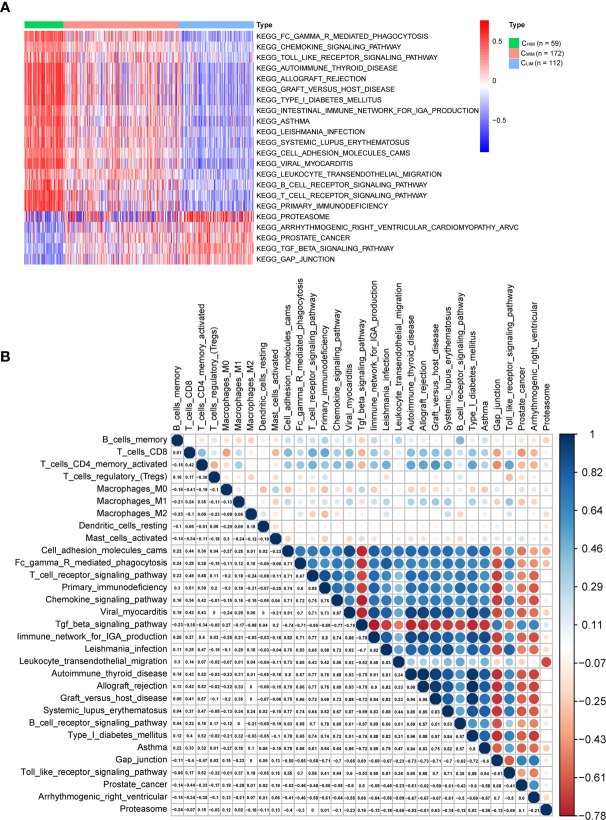
Differential pathways in immune-based subtypes and their correlation with infiltrated immune cells. **(A)** KEGG pathway enrichment was identified by GSVA in three subtypes. Only pathways with significant differences are listed. **(B)** Correlations among these KEGG pathways and infiltrated immune cells in gastric cancer patients.

Activation of pathways within the three immune subtypes was analyzed by GSVA (**Figure 6A** and **Supplementary Figures 5A, B**). C_HIM_ samples were enriched in many immune-related terms, including innate immunity (“toll-like receptor signaling”), adaptive immunity (“B cell receptor signaling pathway”, “T cell receptor signaling pathway”, and “FCγ receptor-mediated phagocytosis”), infection (“Leishmania infection” and “viral myocarditis”), and autoimmune diseases (“systemic lupus erythematosus”, “type I diabetes”, “autoimmune thyroid disease”, “graft versus host disease”, and “asthma”) (**Figure 6A**). Enrichment of these pathways was also observed in the other two datasets (**Supplementary Figures 5A, B**). In C_LIM_ samples of the TCGA cohort, the enriched pathways were related to “TGFβ signaling pathway” and “GAP junction” (**Figure 6A**). The TGFβ signaling pathway has been reported to regulate immunity in tumors negatively (27) and thus might cause a low immunity status. In contrast, there were no enriched terms in the C_MIM_ subgroup.

We then pooled the infiltrated immune cells and pathways that were significantly different between subtypes and performed correlation analyses (**Figure 6B**). CD8^+^ T cells and activated CD4^+^ memory T cells correlated with each other well, and both of them were inversely correlated with the terms “TGFβ signaling pathway” and “GAP junction”, confirming an immune-suppressing role of these pathways in gastric cancer. In addition, CD8^+^ T cells and activated CD4^+^ memory cells were positively correlated with terms “cell adhesion molecules” and “chemokine signal pathway”, in accordance with the previous finding that T cell inflation needs cell adhesion molecules and chemokines (28).

### Construction of PPI Network and Identification of Hub Genes

**Figure 7:**
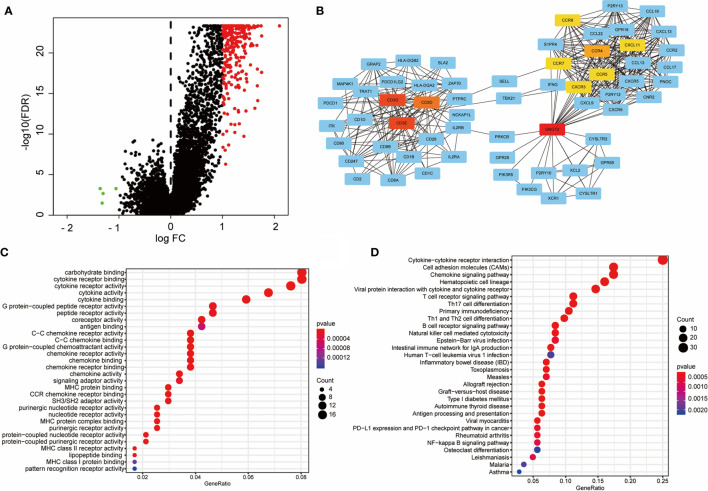
Construction of the PPI network with differentially expressed genes active in C_HIM_ patients. **(A)** Volcano plot of differentially expressed genes between C_HIM_ and C_LIM_ patients. **(B)** PPI network constructed by differentially expressed genes. Only 61 hub genes are shown. Centered genes are highlighted in yellow or red. **(C, D)** Molecular function and KEGG enrichments of hub genes of the PPI network.

We evaluated DEGs and their interaction networks to identify key biological processes in different immune subtypes. Sequencing data of 21,999 mRNAs from C_HIM_ and C_LIM_ samples in the TCGA cohort were compared, and 267 DEGs (3 downregulated and 264 upregulated; |FC| ≥2 and FDR< 0.05) were identified (**Figure 7A**). The upregulated DEGs were then used to construct a PPI network, which included 116 DEGs (confidence cutoff = 0.90). Using Cytoscape 3.7.2 to explore this network, we identified 61 DEGs as hub genes with degree ≥ 10 (**Figure 7B**). These hub genes were mainly enriched in three clusters (1), TCR signaling and their ligands (connected by CD3) (2), chemokines and their ligands (connected by CCR and CXCR families), and (3) G protein-related molecules (connected by GNGT2, **Figure 7B**).

The 61 hub genes were used for enrichment analyses. Molecular function analysis indicated that these genes were highly enriched in ligand-receptor binding-related terms, such as “binding of cytokine receptor”, “G-protein-coupled peptide receptors”, “G-protein-coupled chemoattractant”, “C-C chemokine receptor”, and “MHC proteins”, suggesting that these hub genes play essential roles in cell communications (**Figure 7C**). These results were also validated in the KEGG analysis, from which the receptor-ligand binding pathways were prominent, including “cytokine-cytokine receptor interaction”, “chemokine signaling pathway”, and similar (**Figure 7D**). In addition, the hub genes were also enriched in terms of T cell activation (“T cell receptor signaling pathway”, “Th17 cell differentiation”, “Th1 and Th2 cell differentiation”, and “NK cell-mediated cytotoxicity”), NF-kappa B signaling pathway, and PD-1/PD-L1 checkpoint pathway (**Figure 7D**).

### Association Between Immune Subtypes and Responses to Immunotherapy

**Figure 8:**
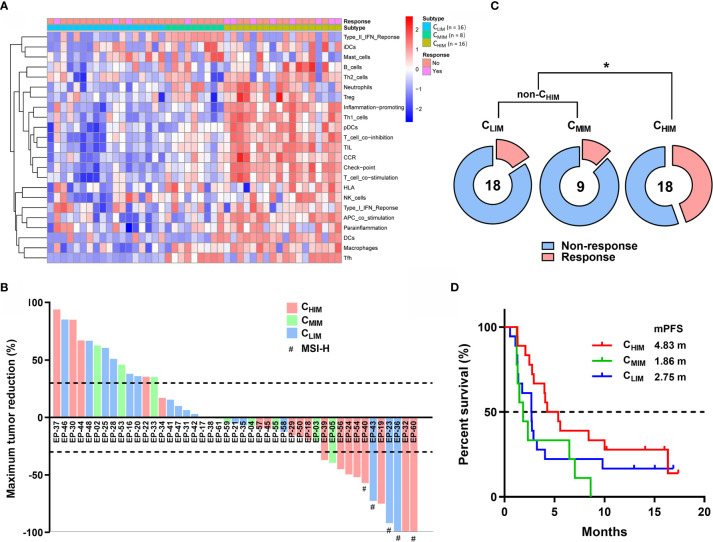
Clinical outcome prediction by immune subtypes in patients treated with ICIs. **(A)** Clustering of patients in PRJEB25780 cohorts. **(B)** Waterfall plot of responses to ICIs (pembrolizumab) according to immune subtypes. The lower dotted line represents tumor reduction of 30% per RECIST and defines partial responses. **(C)** Frequencies of patients with and without response to ICIs between different immune subgroups. **(D)** Kaplan-Meier plot of progression-free survival after treatment with ICIs between immune subtypes. Median progression-free survivals (mPFS) are listed in the panel. *P < 0.05.

Considering the importance of immunotherapy in cancer treatment now, we further examined the association between immune subtypes and responses to ICI using RNA-sequencing and clinical data from PRJEB25780, a clinical trial cohort with metastatic gastric cancer treated with pembrolizumab (**Figure 8A**) (11). Among 18 C_HIM_ patients, 2 achieved complete responses (11.1%), and 6 achieved partial responses (33.3%), resulting in an objective response rate of 44.4% (**Figures 8B, C**). In comparison, the objective response rates were 16.7% (3/18) and 11.1% (1/9) in C_LIM_ and C_MIM_ patients, respectively (**Figures 8B, C**). The response rates between C_HIM_ and non-C_HIM_ were statistically different (P = 0.0277) (**Figure 8C**). Of note, in the 8 C_HIM_ patients with response, only 2 (25%) were MSI-H, while 3/3 (100%) C_LIM_ patients with response were MSI-H, indicating C_HIM_ was likely a predictive factor independent of MSI-H (**Figure 8B**). With immature survival data (9 patients were undergoing treatment and had been followed-up for 10-20 months), C_HIM_ patients achieved a longer median progression-free survival (4.83 months) than non-C_HIM_ patients (1.86 and 2.75 months), although the difference did not reach statistical significance (**Figure 8D**).

## Discussion

We evaluated the immunogenomic features of gastric cancer samples by meta-analysis of consensus expression clustering and proposed three immune subtypes, which differ in tumor purity, richness of stromal cells, and infiltrated immune cells. Such classification has been reported in other types of cancer. For example, three- and six-immune subtype classifications associated with distinct molecular characteristics and clinical outcomes were proposed in colorectal cancer (29) and squamous cell carcinomas (30), respectively. The distinct classification reveals heterogeneity in immune activities of gastric cancer and may help explain why the response to immunotherapy is typically limited to a small subset of patients (31). Therefore, it is essential to stratify gastric cancer patients in an immunological view beyond histology or genomics.

Our classification is simply based on mRNA sequencing data and 29 signatures. Unlike CIBERSORT (32), most of the 29 signatures were derived from tumor sequencing data other than peripheral immune cells or tonsils (12–18). The 29 signatures included a mixture of cell types and functions, so they should be more representative than cells alone, due to that both cell quantities and their functions determine intra-tumor immunity. However, it was limited by lacking more tumor sequencing-based signatures, such as signatures for NTK and MDSC. Like the previously defined “hot tumor” (33), the C_HIM_ subtype is rich in anti-tumor immune cells compared to non-C_HIM_ patients, which is not surprising because our subtyping method was based on immune signatures. Further, data by meta-analysis based on individual patient data revealed that the immune status was associated with patients’ prognosis in gastric cancer. This is consistent with previous findings that immune infiltration, especially CD8^+^ T cell infiltration, is associated with a favorable prognosis (34–36).

Besides infiltrated T cells, other characteristics of “hot tumors” are present in the C_HIM_ subtype (37). They included high expression of immune checkpoints, immune orientation (chemokines and cytokines), and antigen presentation molecules (MHCI and II). In addition, high DNA methylation was also associated with immune activation status, increased tumor mutation and neoantigens, and favorable prognosis in gastric cancer (26). The C_LIM_ subgroup may thus represent “cold tumors”. This provides us with a rationale for tailoring treatment based on immune subtypes. It has been postulated that “hot tumors” responded well to inhibitors of immune checkpoints (such as PD1, PD-L1, CTLA4, TIM3, LAG3, and TIGIT) (33, 38–40), co-stimulatory enhancers (such as OX40, TNFRSF7, CD28, TNFRSF9, and GITR) (33, 38, 41), and microbiome modulators (42). In contrast, these strategies alone are not suitable for “cold tumors” (43). Instead, “cold tumors” may require a combination of routine treatments (such as chemotherapy and radiotherapy) and immunotherapy, with the former destroying parts of tumor cells, promoting tumor antigen release, and heating tumor immunogenicity for improved efficacy of the latter (33). In this aspect, our immune classification may guide clinical treatments of gastric cancer.

An accurate biomarker is essentially significant for gastric cancer because it responds to solo immunotherapy infrequently. dMMR/MSI and EBV are helpful biomarkers but merely account for a small part of patients (11). Tumor mutation burden (TMB) also correlates with responses to ICIs (44), but the definition of high TMB is inconsistent across clinical trials and still under debate (45). Plus, the predictive ability of TMB is not always good in all cancer types (46). ICIs have shown efficacy in patients with high PD-L1 expression, but it is far from being “accurate” as a promising biomarker (8). Following the above discoveries, we further examined the association between the immune subtypes and responses to ICIs. The C_HIM_ subtype showed a higher response to immunotherapy and more prolonged progression-free survival than the rest patients, suggesting that the immune subtype can guide usage of immunotherapeutic drugs. Importantly, this predicting ability was likely independent of MSI status, thus providing a new biomarker for immunotherapy that can compensate MSI for high sensitivity. Since there is only one dataset providing transcriptome information of immune (only pembrolizumab)-treated gastric cancer patients, this conclusion should be drawn cautiously and validated in additional cohorts.

By GSVA, we found “TGFβ signaling pathway” and “GAP junction” were significantly enriched in C_LIM_ tumors and were highly inversely correlated with the tumor-infiltrated T cells. TGFβ is central to immune suppression within the tumor microenvironments by inhibiting antigen presentation cells and effector T cells (27). In contrast, the roles of GAP junction in tumor immunity were rarely reported and are worth further exploration in gastric cancer.

Furthermore, we found some characteristics beyond the transcriptome data. Although the TMB was comparable in C_HIM_ and C_LIM_ tumors, several genes, including *ARID1A*, *TP53*, and *SYNE2*, were differentially mutated in these subtypes. *ARID1A* mutation is a biomarker for immune checkpoint blockade therapy in several types of cancers (47), and it shapes cancer immune phenotype by dMMR (48) and defining cancer interferon responsiveness and immune evasion (49). *TP53* mutation in cancers can affect the recruitment and activity of myeloid and T cells, allowing immune evasion and tumor progression (50). In gastric cancer, *TP53* mutation resulted in depressed immune activities (51) and inadequate responses to ICIs in patients with HLA-B62 supertype (52). Of note, *TP53* mutation was reported to increase immune checkpoint expression and activate T-effector and interferon-γ signature in lung adenocarcinoma (53). Lung cancer patients with *TP53* mutation, especially those with co-mutation of *KRAS*, showed remarkable clinical benefit to PD-1 inhibitors (53).

Based on RPPA data, several proteins were identified to be over-expressed in C_LIM_ subgroups, which might be the cause of immune inactivity. Among them, β-catenin is an intracellular signal transducer of the Wnt pathway, which induces immune evasion and is negatively associated with CD8^+^ T cell infiltration at the tumor site (54, 55). E-cadherin is an epithelial marker and forms a complex with β-catenin to maintain cell-to-cell adhesion (56). Its downregulation may increase permeability and lead to immune cell infiltration, which has been pathologically shown in gallbladder cancer (57) and colorectal cancer (58). Cyclin E1 is a cell cycle regulator, and cyclin E1-driven ovarian cancer is characterized by decreased cancer immunity mediated by activated polyamine synthesis (59). In contrast, several proteins, including PREX1, LCK, PD-L1, transglutaminase, and cleaved caspase 7, were elevated in the C_HIM_ subgroup, which might be predictors of “hot tumor”. LCK and transglutaminase are essential for T cell activation (60), while PREX1 and PD-L1 are both co-inhibitory immune checkpoints (61), which are potential markers of “hot tumors”. Caspase-7 is an apoptotic effector with pro-inflammatory abilities (62). Under inflammation, it is cleaved by inflammasomes and promotes inflammation (62). Therefore, we believe that the cleaved caspase-7 may represent an inflammation (“hot”) status in tumor microenvironments. However, the roles of transglutaminase, PREX1, and caspase-7 in tumor immunity are rarely studied, and further investigation would be valuable.

Several limitations remained in this study. First, due to the restriction of tumor sequencing-based immune-related signatures, the gene sets used in this study could not cover all immune cell types and functions. Clustering with a broader and more comprehensive range of immune-related signatures may result in better predictive values. Second, this is a retrospective analysis based on published datasets. For an application in clinical practice, a prospective study is needed to avoid bias. Third, methods used to verify the subtype features, such as CIBERSORT and KEGG, were indirectly based on bulk RNA sequencing. Validations at single-cell levels, such as multiple immunofluorescence staining, mass cytometry, and single-cell sequencing, should be considered in future studies.

In conclusion, the immune signature-based classification stratified gastric cancer patients with different clinical outcomes, characteristics of tumor immunity, mutation landscape, epigenetic patterns, and pathway activation. This system is clinically feasible and has potential clinical implications for gastric cancer treatment.

## Data Availability Statement

The original contributions presented in the study are included in the article/**Supplementary Material**. Further inquiries can be directed to the corresponding author.

## Author Contributions

All authors contributed to the article and approved the submitted version. LL designed this study. SL performed the main parts of the analysis and wrote the manuscript. JG, QX, XZ, MH, XD, and KH helped with data collecting and analysis. LL revised the manuscript.

## Funding

This work was supported by the National Natural Science Foundation of China (81172487 to LL and 81500092 to SL), Natural Science Foundation of Shandong Province (ZR201702180008 to LL), and Foundation of Shandong University Clinical Research Center (2020SDUCRCC011).

## Conflict of Interest

The authors declare that the research was conducted in the absence of any commercial or financial relationships that could be construed as a potential conflict of interest.
