# Supplementary material for: A Signature-Based Classification of Gastric Cancer That Stratifies Tumor Immunity and Predicts Responses to PD-1 Inhibitors
Source: Front Immunol. 2021 Jun 11;12:693314. doi: 10.3389/fimmu.2021.693314 (PMC8232232; doi:10.3389/fimmu.2021.693314)
Supplement: Supplementary file 1 [file DataSheet_1.docx]

Supplementary Material

# Supplementary Figures


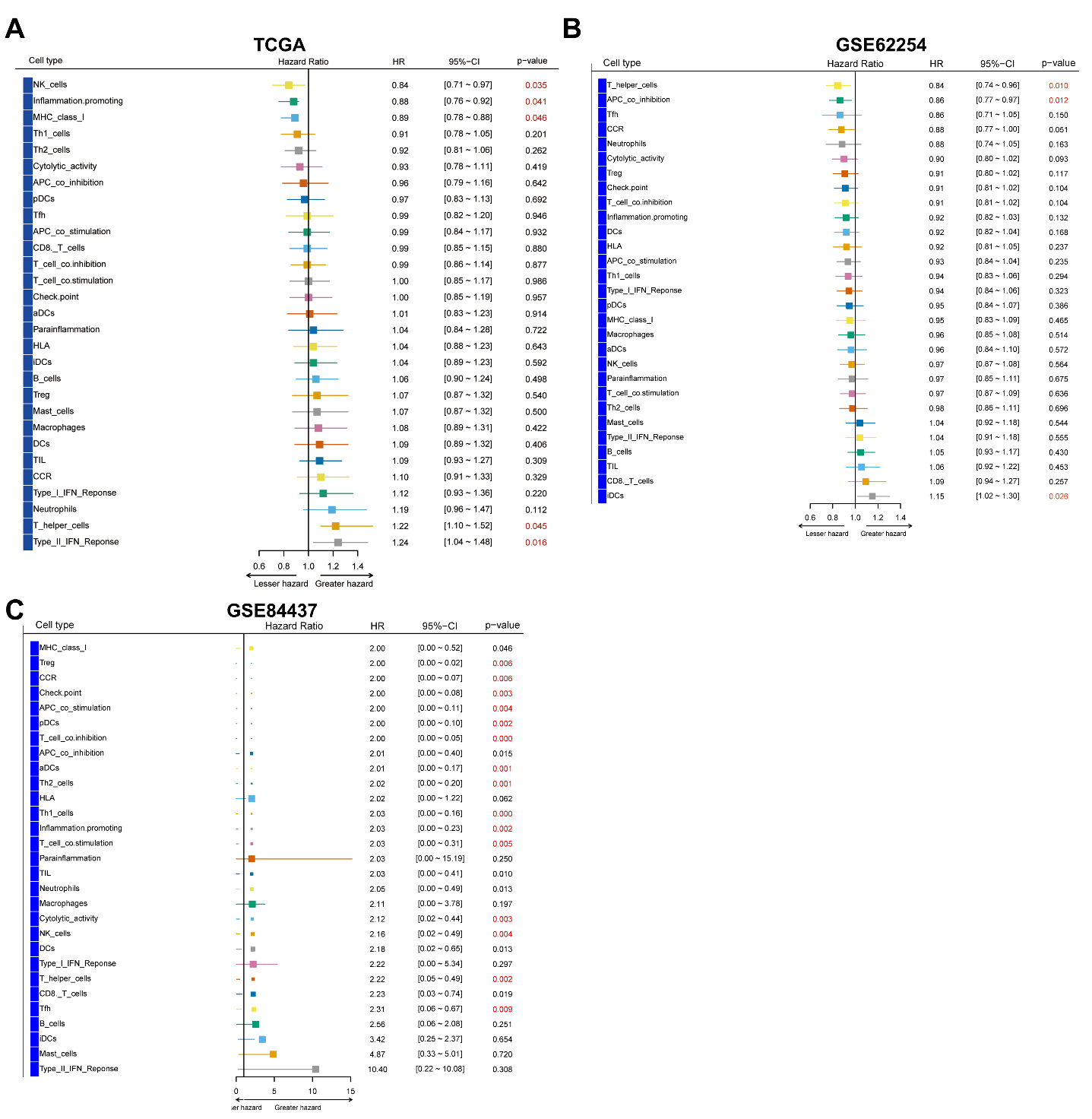


**Supplementary Figure 1.** Correlations between immune signatures and overall survivals in GC from TCGA(A), GSE62254 (B), and GSE84437 (C) cohorts. HRs and 95% CIs were calculated by univariate cox regression. Significant p values (< 0.05) were marked in red. **p* < 0.05, ***p* < 0.01, ****p* < 0.001, *****p* < 0.0001.


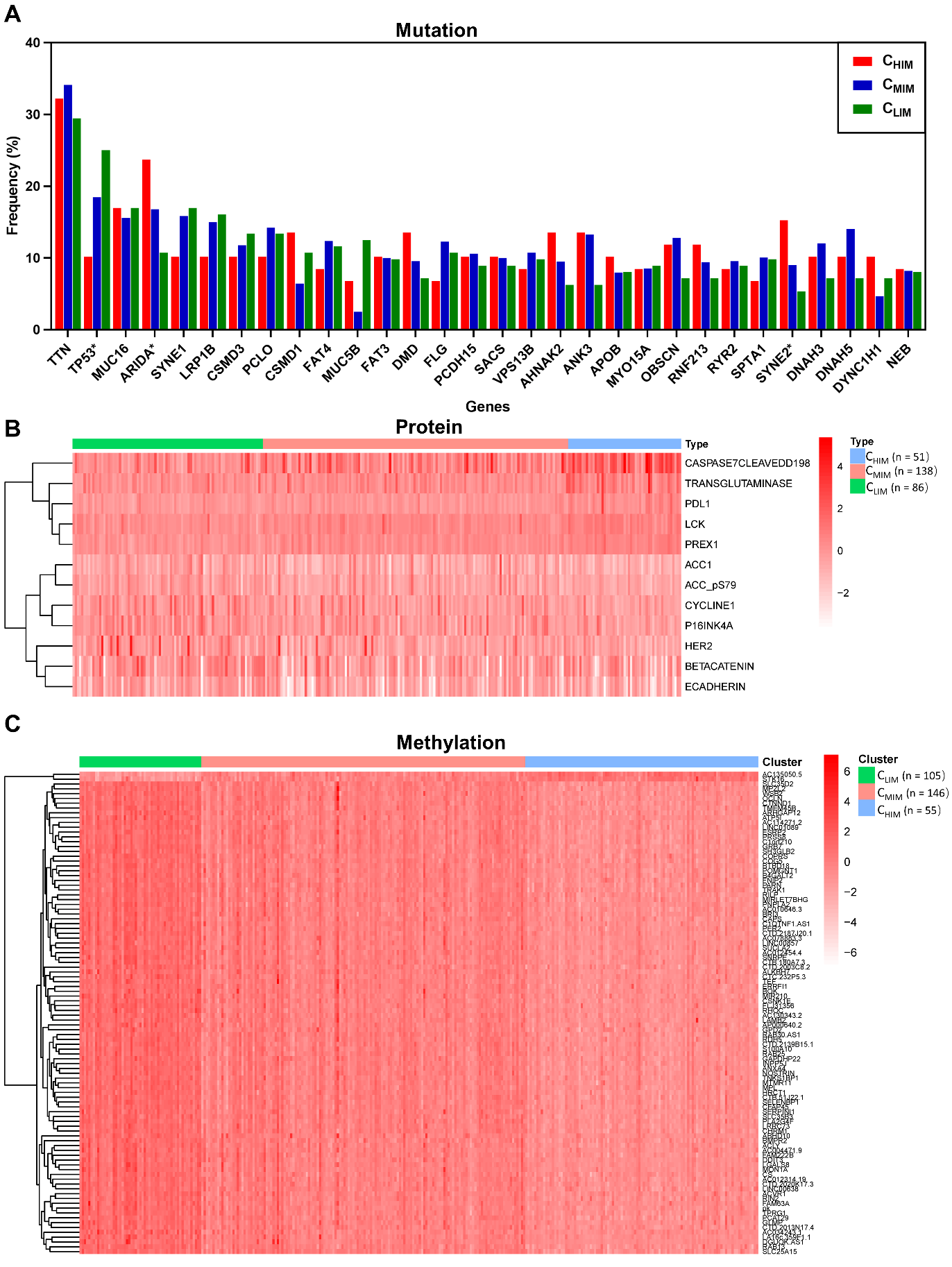


**Supplementary Figure 2.** Genomic, epigenomic, and proteomic features of different immune subtypes. (A) Frequencies of mutations of the top 30 mutated genes in 3 immune subtypes. (B) Differentially expressed proteins in 3 immune subgroups. (C) Top 100 differential methylated genes in 3 immune subgroups. *p < 0.05.


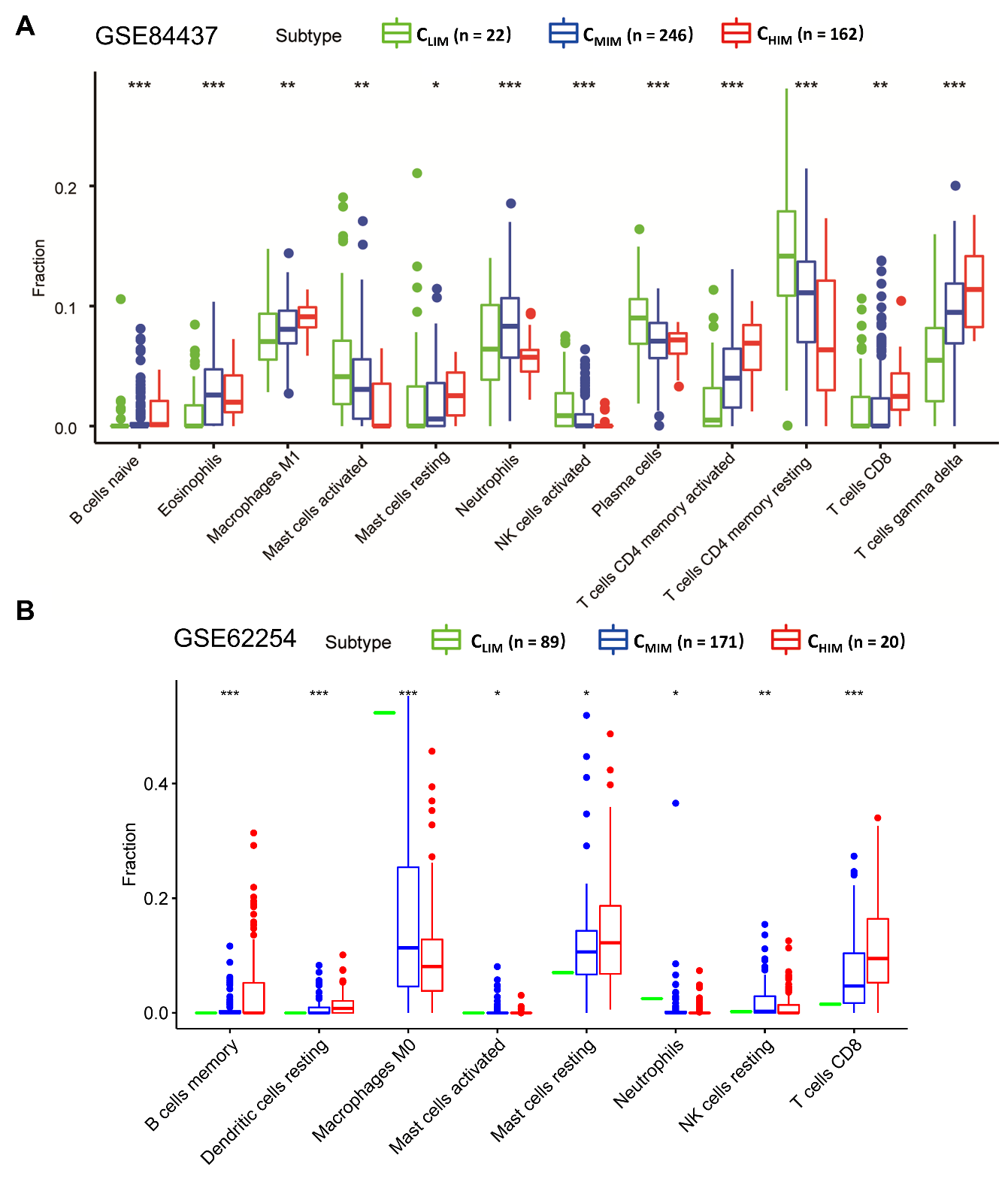


**Supplementary Figure 3.** Fractions of tumor-infiltrated immune cells in three immune subtypes in the GSE84437 (A) and GSE62254 (B) cohorts. Only cells with significant differences among the subtypes were shown. **p* < 0.05, ***p* < 0.01, ****p* < 0.001.


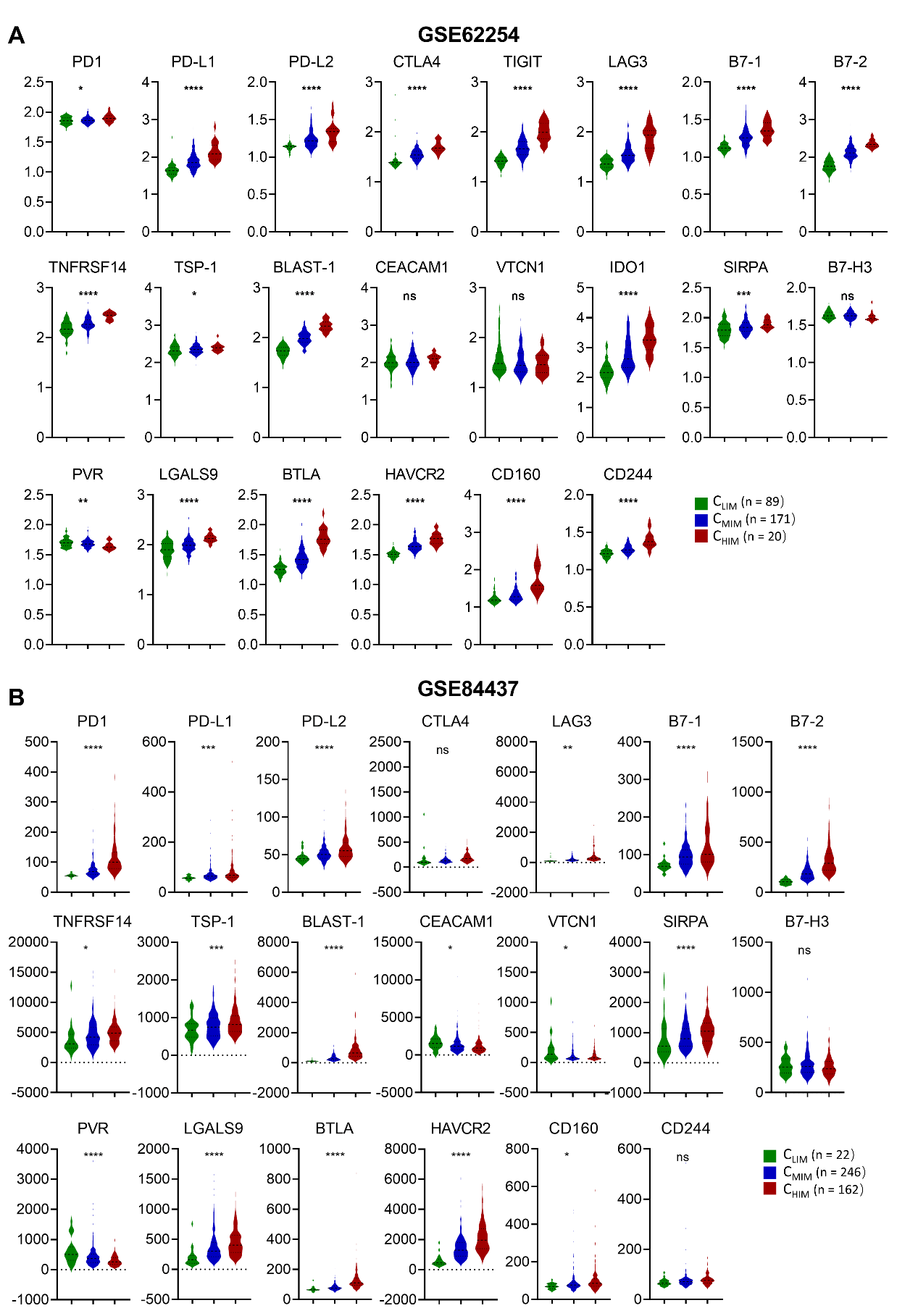


**Supplementary Figure 4.** Levels of co-inhibitory molecules in three immune subtypes in GSE62254 (A) and GSE84437 (B) cohorts. *P*-values with significance were marked in red.


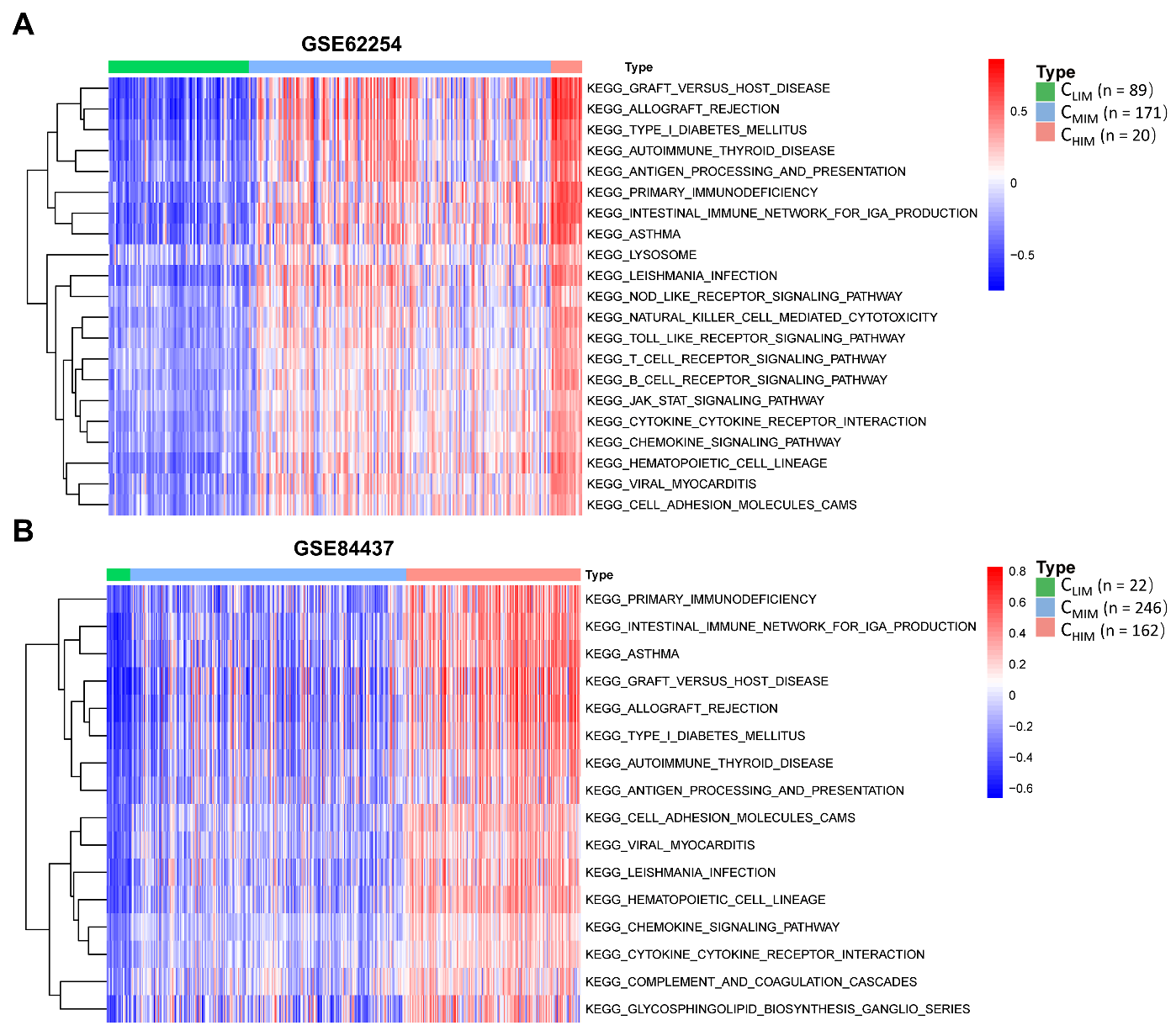


**Supplementary Figure 5.** Differential pathway enrichment identified by GSVA in three subtypes in GSE62254 (A) and GSE84437 (B) cohorts. Only pathways with significant differences were listed.

| **Supplementary Table 1.** Composition of the 29 immune signatures used for subtype clustering. | |
| --- | --- |
| **Gene set** | **Genes** |
| aDCs | CD83, LAMP3, CCL1 |
| APC co-inhibition | C10orf54, CD274, LGALS9, PDCD1LG2, PVRL3 |
| APC co-stimulation | CD40, CD58, CD70, ICOSLG, SLAMF1, TNFSF14, TNFSF15, TNFSF18, TNFSF4, TNFSF8, TNFSF9 |
| B cells | BACH2, BANK1, BLK, BTLA, CD79A, CD79B, FCRL1, FCRL3, HVCN1, RALGPS2 |
| CCR | CCL16, TPO, TGFBR2, CXCL2, CCL14, TGFBR3, IL11RA, CCL11, IL4I1, IL33, CXCL12, CXCL10, BMPER, BMP8A, CXCL11, IL21R, IL17B, TNFRSF9, ILF2, CX3CR1, CCR8, TNFSF12, CSF3, TNFSF4, BMP3, CX3CL1, BMP5, CXCR2, TNFRSF10D, BMP2, CXCL14, CCL28, CXCL3, BMP6, CCL21, CXCL9, CCL23, IL6, TNFRSF18, IL17RD, IL17D, IL27, CCL7, IL1R1, CXCR4, CXCR2P1, TGFB1I1, IFNGR1, IL9R, IL1RAPL1, IL11, CSF1, IL20RA, IL25, TNFRSF4, IL18, ILF3, CCL20, TNFRSF12A, IL6ST, CXCL13, IL12B, TNFRSF8, IL6R, BMPR2, IFNE, IL1RAPL2, IL3RA, BMP4, CCL24, TNFSF13B, CCR4, IL2RA, IL32, TNFRSF10C, IL22RA1, BMPR1A, CXCR5, CXCR3, IFNA8, IL17REL, IFNB1, IFNAR1, TNFRSF1B, CCL17, IFNL1, IL16, IL1RL1, ILK, CCL25, ILDR2, CXCR1, IL36RN, IL34, TGFB1, IFNG, IL19, ILKAP, BMP2K, CCR10, ILDR1, EPO, CCR7, IL17C, IL23A, CCR5, IL7, EPOR, CCL13, IL2RG, IL31RA, TNFAIP6, IFNL2, BMP1, IL12RB1, TNFAIP8, IL4R, TNFRSF6B, TNFAIP8L1, TNFRSF10B, IFNL3, CCL5, CXCL6, CXCL1, CCR3, TNFSF11, CSF1R, IL21, IL1RAP, IL12RB2, CCL1, IL17RA, CCR1, IL1RN, TNFRSF11B, TNFRSF14, IL13, IL2RB, BMP8B, CCL2, IL24, IL18RAP, TGFBI, TNFSF10, TNFRSF11A, CXCL5, IL5RA, TNFSF9, IL1RL2, TNFRSF13C, IL36G, IL15RA, TNFRSF21, CXCL8, IL22RA2, TNFAIP8L2, IL18R1, IFNLR1, CXCR6, CCL3L3, TNFRSF1A, IL17RE, IFNGR2, IL17RC, TNFAIP8L3, ILVBL, TGFBRAP1, CCL4L1, CSF2RA, CCRN4L, CCL26, TNFAIP1, CCRL2, IFNA10, TNFRSF17, IFNA13, IL20, IL18BP, CCL3L1, TNFSF12-TNFSF13, IL5, IL23R, IL26, TNF, TGFA, CSF2, IL1F10, CXCL17, TNFSF13, IFNA4, IL37, IL12A, IL7R, IFNA1, IL1A, IL4, IL2, CCL22, CSF3R, IL10, IFNK, TGFB2, IL1R2, IL1B, IL17F, IL27RA, IL15, TNFSF8, IL36B, XCL1, CXCL16, TNFRSF19, IL3, CCL3, IFNA2, BMPR1B, IFNA21, TNFSF18, CCL8, IL17RB, TNFRSF25, IL22, IL10RB, IFNAR2, CCL18, IFNA16, CSF2RB, IL36A, TNFAIP3, IL13RA2, IL13RA1, CCR9, TNFRSF10A, IFNA7, IFNW1, XCL2, TNFSF14, CCR2, BMP15, BMP10, CCL15-CCL14, TGFBR1, IFNA5, BMP7, IFNA14, IL20RB, IL10RA, IFNA17, CCR6, TGFB3, CCL15, CCL4, CCL27, TNFRSF13B, TNFAIP2, IL31, IL17A, TNFSF15, CCL19, IFNA6, IL9 |
| CD8+ T cells | CD8A |
| Check-point | IDO1, LAG3, CTLA4, TNFRSF9, ICOS, CD80, PDCD1LG2, TIGIT, CD70, TNFSF9, ICOSLG, KIR3DL1, CD86, PDCD1, LAIR1, TNFRSF8, TNFSF15, TNFRSF14, IDO2, CD276, CD40, TNFRSF4, TNFSF14, HHLA2, CD244, CD274, HAVCR2, CD27, BTLA, LGALS9, TMIGD2, CD28, CD48, TNFRSF25, CD40LG, ADORA2A, VTCN1, CD160, CD44, TNFSF18, TNFRSF18, BTNL2, C10orf54, CD200R1, TNFSF4, CD200, NRP1 |
| Cytolytic activity | PRF1, GZMA |
| DCs | CCL17, CCL22, CD209, CCL13 |
| HLA | HLA-E, HLA-DPB2, HLA-C, HLA-J, HLA-DQB1, HLA-DQB2, HLA-DQA2, HLA-DQA1, HLA-A, HLA-DMA, HLA-DOB, HLA-DRB1, HLA-H, HLA-B, HLA-DRB5, HLA-DOA, HLA-DPB1, HLA-DRA, HLA-DRB6, HLA-L, HLA-F, HLA-G, HLA-DMB, HLA-DPA1 |
| iDCs | CD1A, CD1E |
| Inflammation-promoting | CCL5, CD19, CD8B, CXCL10, CXCL13, CXCL9, GNLY, GZMB, IFNG, IL12A, IL12B, IRF1, PRF1, STAT1, TBX21 |
| Macrophages | C11orf45, CD68, CLEC5A, CYBB, FUCA1, GPNMB, HS3ST2, LGMN, MMP9, TM4SF19 |
| Mast cells | CMA1, MS4A2, TPSAB1 |
| MHC class I | B2M, HLA-A, TAP1 |
| Neutrophils | EVI2B, HSD17B11, KDM6B, MEGF9, MNDA, NLRP12, PADI4, SELL, TRANK1, VNN3 |
| NK cells | KLRC1, KLRF1 |
| Parainflammation | CXCL10, PLAT, CCND1, LGMN, PLAUR, AIM2, MMP7, ICAM1, MX2, CXCL9, ANXA1, TLR2, PLA2G2D, ITGA2, MX1, HMOX1, CD276, TIRAP, IL33, PTGES, TNFRSF12A, SCARB1, CD14, BLNK, IFIT3, RETNLB, IFIT2, ISG15, OAS2, REL, OAS3, CD44, PPARG, BST2, OAS1, NOX1, PLA2G2A, IFIT1, IFITM3, IL1RN |
| pDCs | CLEC4C, CXCR3, GZMB, IL3RA, IRF7, IRF8, LILRA4, PHEX, PLD4, PTCRA |
| T cell co-inhibition | BTLA, C10orf54, CD160, CD244, CD274, CTLA4, HAVCR2, LAG3, LAIR1, TIGIT |
| T cell co-stimulation | CD2, CD226, CD27, CD28, CD40LG, ICOS, SLAMF1, TNFRSF18, TNFRSF25, TNFRSF4, TNFRSF8, TNFRSF9, TNFSF14 |
| T helper cells | CD4 |
| Tfh | PDCD1, CXCL13, CXCR5 |
| Th1 cells | IFNG, TBX21, CTLA4, STAT4, CD38, IL12RB2, LTA, CSF2 |
| Th2 cells | PMCH, LAIR2, SMAD2, CXCR6, GATA3, IL26 |
| TIL | ITM2C, CD38, THEMIS2, GLYR1, ICOS, F5, TIGIT, KLRD1, IRF4, PRKCQ, FCRL5, SIRPG, LPXN, IL2RG, CCL5, LCK, TRAF3IP3, CD86, MAL, LILRB1, DOK2, CD6, PAG1, LAX1, PLEK, PIK3CD, SLAMF1, XCL1, GPR171, XCL2, TBX21, CD2, CD53, KLHL6, SLAMF6, CD40, SIT1, TNFRSF4, CD79A, CD247, LCP2, CD3D, CD27, SH2D1A, FYB, ARHGAP30, ACAP1, CST7, CD3G, IL2RB, CD3E, FCRL3, CORO1A, ITK, TCL1A, CYBB, CSF2RB, IKZF1, NCF4, DOCK2, CCR2, PTPRC, PLAC8, NCKAP1L, IL7R, 6-Sep, CD28, STAT4, CD8A, LY9, CD48, HCST, PTPRCAP, SASH3, ARHGAP25, LAT, TRAT1, IL10RA, PAX5, CCR7, DOCK11, PARVG, SPNS1, CD52, HCLS1, ARHGAP9, GIMAP6, PRKCB, MS4A1, GPR18, TBC1D10C, GVINP1, P2RY8, EVI2B, VAMP5, KLRK1, SELL, MPEG1, MS4A6A, ARHGAP15, MFNG, GZMK, SELPLG, TARP, GIMAP7, FAM65B, INPP5D, ITGA4, MZB1, GPSM3, STK10, CLEC2D, IL16, NLRC3, GIMAP5, GIMAP4, IFFO1, CFH, PVRIG, CFHR1 |
| Treg | IL12RB2, TMPRSS6, CTSC, LAPTM4B, TFRC, RNF145, NETO2, ADAT2, CHST2, CTLA4, NFE2L3, LIMA1, IL1R2, ICOS, HSDL2, HTATIP2, FKBP1A, TIGIT, CCR8, LTA, SLC35F2, IL21R, AHCYL1, SOCS2, ETV7, BCL2L1, RRAGB, ACSL4, CHRNA6, BATF, LAX1, ADPRH, TNFRSF4, ANKRD10, CD274, CASP1, LY75, NPTN, SSTR3, GRSF1, CSF2RB, TMEM184C, NDFIP2, ZBTB38, ERI1, TRAF3, NAB1, HS3ST3B1, LAYN, JAK1, VDR, LEPROT, GCNT1, PTPRJ, IKZF2, CSF1, ENTPD1, TNFRSF18, METTL7A, KSR1, SSH1, CADM1, IL1R1, ACP5, CHST7, THADA, CD177, NFAT5, ZNF282, MAGEH1 |
| Type I IFN Reponse | DDX4, IFIT1, IFIT2, IFIT3, IRF7, ISG20, MX1, MX2, RSAD2, TNFSF10 |
| Type II IFN Reponse | GPR146, SELP, AHR |

| **Supplementary Table 2.** Distribution of clinical features in three immune subtypes. | | | | | | | | | | | | | |
| --- | --- | --- | --- | --- | --- | --- | --- | --- | --- | --- | --- | --- | --- |
|  |  | TCGA | | | | GSE62254 | | | | GSE84437 | | | |
|  |  | C_LIM_ | C_MIM_ | C_HIM_ | *P* value | C_LIM_ | C_MIM_ | C_HIM_ | *P* value | C_LIM_ | C_MIM_ | C_HIM_ | *P* value |
| Age | ≤65 | 43 (0.38) | 80 (0.47) | 29 (0.49) | 0.2883 | 45 (0.51) | 116 (0.61) | 11 (0.55) | 0.2705 | 18 (0.82) | 158 (0.63) | 107 (0.66) | 0.2159 |
|  | >65 | 69 (0.62) | 92 (0.53) | 30 (0.51) |  | 44 (0.49) | 75 (0.39) | 9 (0.45) |  | 4 (0.18) | 91 (0.37) | 55 (0.34) |  |
| Gender | Male | 75 (0.67) | 106 (0.62) | 35 (0.59) | 0.5390 | 67 (0.75) | 119 (0.62) | 13 (0.65) | 0.1005 | 14 (0.64) | 176 (0.71) | 106 (0.65) | 0.4747 |
|  | Female | 37 (0.33) | 66 (0.38) | 24 (0.41) |  | 22 (0.25) | 72 (0.38) | 7 (0.35) |  | 8 (0.36) | 73 (0.29) | 56 (0.35) |  |
| Stage | Stage I | 23 (0.21) | 23 (0.15) | 4 (0.07) | 0.1621 | 9 (0.10) | 19 (0.10) | 2 (0.10) | 0.8052 | NA | NA | NA | NA |
|  | Stage II | 34 (0.32) | 51 (0.32) | 17 (0.3) |  | 34 (0.39) | 57 (0.3) | 5 (0.25) |  | NA | NA | NA |  |
|  | Stage III | 37 (0.35) | 67 (0.43) | 31 (0.54) |  | 25 (0.28) | 62 (0.33) | 8 (0.4) |  | NA | NA | NA |  |
|  | Stage IV | 13 (0.12) | 16 (0.1) | 5 (0.09) |  | 20 (0.23) | 52 (0.27) | 5 (0.25) |  | NA | NA | NA |  |
| T stage | T1 | 11 (0.1) | 8 (0.05) | 0 (0.00) | 0.0943 | 0  (0.00) | 0 (0.00) | 0 (0.00) | 0.0729 | 0  (0.00) | 7 (0.03) | 4 (0.02) | 0.0533 |
|  | T2 | 25 (0.23) | 38 (0.23) | 11 (0.19) |  | 63 (0.72) | 113 (0.59) | 10 (0.5) |  | 3 (0.14) | 17 (0.07) | 18 (0.11) |  |
|  | T3 | 54 (0.49) | 73 (0.44) | 30 (0.51) |  | 17 (0.19) | 65 (0.34) | 9 (0.45) |  | 10 (0.45) | 52 (0.21) | 30 (0.19) |  |
|  | T4 | 21 (0.19) | 46 (0.28) | 18 (0.31) |  | 8 (0.09) | 12 (0.06) | 1 (0.05) |  | 9 (0.41) | 173 (0.69) | 110 (0.68) |  |
| N stage | N0 | 37 (0.35) | 51 (0.31) | 14 (0.25) | 0.6723 | 13 (0.15) | 23 (0.12) | 2 (0.10) | 0.7348 | 6 (0.27) | 48 (0.19) | 26 (0.16) | 0.6925 |
|  | N1 | 26 (0.24) | 45 (0.28) | 18 (0.32) |  | 43 (0.48) | 79 (0.41) | 9 (0.45) |  | 9 (0.41) | 102 (0.41) | 77 (0.48) |  |
|  | N2 | 25 (0.23) | 36 (0.22) | 10 (0.18) |  | 20 (0.22) | 53 (0.28) | 7 (0.35) |  | 6 (0.27) | 81 (0.33) | 45 (0.28) |  |
|  | N3 | 19 (0.18) | 31 (0.19) | 15 (0.26) |  | 13 (0.15) | 36 (0.19) | 2 (0.10) |  | 1 (0.05) | 18 (0.07) | 14 (0.09) |  |
| M stage | M0 | 101 (0.94) | 151 (0.93) | 52 (0.93) | 0.9616 | 84 (0.94) | 174 (0.91) | 15 (0.75) | 0.0236 | NA | NA | NA | NA |
|  | M1 | 7 (0.06) | 12 (0.07) | 4 (0.07) |  | 5 (0.06) | 17 (0.09) | 5 (0.25) |  | NA | NA | NA |  |
| MSI/  MMR | MSS/  MMRp | 100 (0.89) | 134 (0.78) | 49 (0.83) | 0.0590 | 13 (0.15) | 52 (0.27) | 2 (0.1) | 0.0241 | NA | NA | NA | NA |
|  | MSI-H/  MMRd | 12 (0.11) | 37 (0.22) | 10 (0.17) |  | 76 (0.85) | 139 (0.73) | 18 (0.9) |  |  |  |  |  |
| H pylori | No | 51 (0.93) | 58 (0.89) | 18 (0.75) | 0.0756 | 29 (0.71) | 42 (0.52) | 1 (0.2) | 0.0333 | NA | NA | NA | NA |
|  | Yes | 4 (0.07) | 7 (0.11) | 6 (0.25) |  | 12 (0.29) | 39 (0.48) | 4 (0.8) |  |  |  |  |  |
| EBV | Positive | 0  (0.00) | 9 (0.05) | 14 (0.24) | <0.0001 | 0  (0.00) | 9 (0.05) | 9 (0.53) | <0.0001 | NA | NA | NA | NA |
|  | Negative | 112 (1) | 163 (0.95) | 45 (0.76) |  | 81 (1.00) | 168 (0.95) | 8 (0.47) |  |  |  |  |  |
| Race | White | 68 (0.73) | 109 (0.78) | 40 (0.77) | 0.4641 | Cohorts in South Korea | | | | Cohorts in South Korea | | | |
|  | Asian | 25 (0.27) | 30 (0.22) | 12 (0.23) |  |  |  |  |  |  |  |  |  |
|  | Black or African American | 3 (0.03) | 7 (0.05) | 0  (0.00) |  |  |  |  |  |  |  |  |  |
|  | Native Hawaiian or other Pacific Islander | 1 (0.01) | 0 (0.00) | 0 (0.00) |  |  |  |  |  |  |  |  |  |

MSI, microsatellite instability; MMR, mismatch repair; MMRd, MMR-deficient; MMRp, MMR-proficient; NA, not available.
